# IGFBP7 remodels the tumor microenvironment of esophageal squamous cell carcinoma by activating the TGFβ1/SMAD signaling pathway

**DOI:** 10.3892/ol.2022.13621

**Published:** 2022-12-06

**Authors:** Xiuqing Li, Ji Zhang, Youshan Wu, Chuntao Ma, Dongying Wei, Lijuan Pan, Liangliang Cai

Oncol Lett 24: 251, 2022; DOI: 10.3892/ol.2022.13371

Following the publication of the above article, an interested reader drew to the authors’ attention that there appeared to be mismatches between the western blotting data shown in Fig. 1C as it appeared on p. 3, and the semiquantification of the same data shown in the histogram on the right-hand side of the figure.

The authors have consulted their original data, and realize that errors were made with the semiquantification of the data; therefore, a revised version of Fig. 1, showing the correct data in the histogram in Fig. 1C, is shown below. All the authors approve of the publication of this corrigendum, and the authors are grateful to the Editor of *Oncology Letters* for granting them the opportunity to publish this. The authors are grateful to the interested reader for drawing this matter to their attention, and also apologize to the readership for any inconvenience caused.

## Figures and Tables

**Figure 1.**
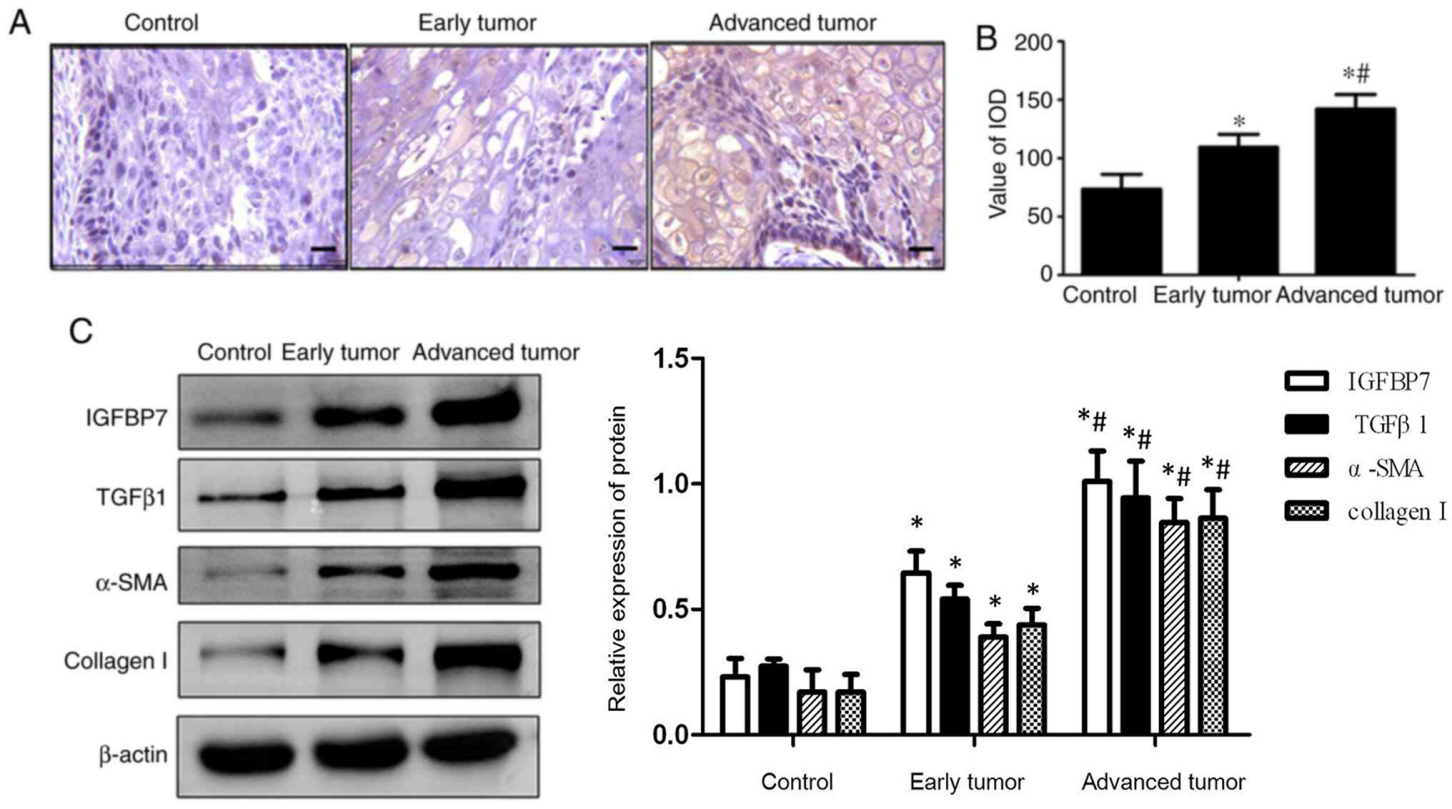
Expression of IGFBP7, TGFβ1, α-SMA, and collagen I in esophageal squamous cell carcinoma. (A) IGFBP7 expression was examined by immunohistochemistry staining (scale bar, 50 µm). (B) IOD value of the positive-brown particles was calculated. (C) Expression of IGFBP7, TGFβ1, α-SMA, and collagen I was examined by western blotting. β-actin served as an internal control (n=15). *P<0.05 vs. the control group; ^#^P<0.05 vs. the early tumor group. IGFBP7, insulin-like growth factor-binding protein-7; TGFβ1, transforming growth factor-β1; α-SMA, α-smooth muscle actin.

